# Supplementary material for: The Quality, Reliability, and Accuracy of Videos Regarding Exercises and Management for Dysphagia in Pediatric Populations Uploaded on YouTube
Source: Children (Basel). 2022 Oct 4;9(10):1514. doi: 10.3390/children9101514 (PMC9600706; doi:10.3390/children9101514)
Supplement: Supplementary file 1 [file children-09-01514-s001.zip › children-1938333-supplementary.pdf]

Supplementary Table S1. The link of YouTube videos regarding exercises and management for dysphagia in pediatric population

Number	Link	Title of Youtube Videos
1	https://www.youtube.com/watch?v=00wP5Ivyb8M	#e201 Preview: Developing Critical Thinking Skills in Pediatric Dysphagia - Part 1
2	https://www.youtube.com/watch?v=uIMngL07UF0	#e202 Preview: Developing Critical Thinking Skills in Pediatric Dysphagia - Part 2
3	https://www.youtube.com/watch?v=xNbjU4Pg46M	#e203 Preview: Developing Critical Thinking Skills in Pediatric Dysphagia - Part 3
4	https://www.youtube.com/watch?v=tzTMq8ZvB9A	5 Ways to Use Throat Scope in Pediatric Feeding Therapy
5	https://www.youtube.com/watch?v=QOcBCd-4I4c	6 Oral Motor Exercises for Babies - Oral Stimulation- Speech Development- Feeding Development
6	https://www.youtube.com/watch?v=42Uk3SCQ1Ac	Assessment and Treatment of Pediatric Feeding Disorders
7	https://www.youtube.com/watch?v=IWVT0AczzAc&list=PLp0zkRPXYYRfor8MoG71-JZnnb6HXy_qM&index=4	Autism and Food- Autistic Toddler Feeding Routine
8	https://www.youtube.com/watch?v=bKViwlqWil4	Clinical Services for the Pediatric Feeding Population
9	https://www.youtube.com/watch?v=bwcdQgwG3zM	Dr Browns Medical Webinar - The Pediatric Intensive Care Unit Dysphagia Management in the Trenches
10	https://www.youtube.com/watch?v=h3oSy5Ljupc	Dysphagia in Children
11	https://www.youtube.com/watch?v=wVaNIriHLk4	Dysphagia in Children with Down Syndrome
12	https://www.youtube.com/watch?v=agURHCdheY0	Dysphagia in the paediatric population
13	https://www.youtube.com/watch?v=JWYL8i2tAOA	Dysphagia training 2.0
14	https://www.youtube.com/watch?v=Z9ODy2P8uZY	Dysphagia Treatment Video 2
15	https://www.youtube.com/watch?v=gzP8SA	Dysphagia, GERD, Hiatal Hernia -

	IXB7c	Medical-Surgical (Med-Surg) - Gastrointestinal (GI) System
16	https://www.youtube.com/watch?v=nmzxyPxqXkY	Evaluating Feeding and Swallowing Disorders in Infants, Children
17	https://www.youtube.com/watch?v=0mGbOeuuuB8	Feeding and Swallowing - Feeding Therapy Sessions - The Children's Hospital of Philadelphia (3 of 6)
18	https://www.youtube.com/watch?v=MI919Yjey34	Feeding and Swallowing Center - Introduction - The Children's Hospital of Philadelphia (1 of 6)
19	https://www.youtube.com/watch?v=2kr8fnXj58k	Feeding Matters - Pediatric Feeding Disorder
20	https://www.youtube.com/watch?v=wwArSmM394g	Feeding Therapy | How To Introduce New Foods | Pediatric Occupational Therapy Tips
21	https://www.youtube.com/watch?v=vAoVRuGavxs	Feeding Therapy at Home | Our Toddler's Oral Feeding Routine
22	https://www.youtube.com/watch?v=J1VVFRnWGoQ	Feeding, Eating & Swallowing for Children with Disabilities by OT Mentorship
23	https://www.youtube.com/watch?v=7D_zeDZTSHI	Functional Education : muscular and swallowing exercises
24	https://www.youtube.com/watch?v=PZwj6P9pNjw	How to Improve Suck Reflex in Babies
25	https://www.youtube.com/watch?v=x1ibGoeFUbI	How we apply feeding therapy at home
26	https://www.youtube.com/watch?v=eWCBiIQ1oGw	Infant Feeding Development | OT Miri
27	https://www.youtube.com/watch?v=n_f0N0tJUeo	Infant Feeding Tips from a Pediatric Occupational Therapist
28	https://www.youtube.com/watch?v=WCinLfKw3WA	Integrative Pediatric Feeding Therapy
29	https://www.youtube.com/watch?v=74lakvAfwuM	Management of Pediatric Feeding and Swallowing
30	https://www.youtube.com/watch?v=GnQEm2HnlwI	Marianjoy's Pediatric Feeding and Swallowing Evaluation Clinic
31	https://www.youtube.com/watch?v=2_MvSzEE3GE	MeBe Learning: All About Pediatric Feeding Disorders
32	https://www.youtube.com/watch?v=mcmp9	Mechanism of Swallowing, Animation in

	s2ra2c	Child model
33	https://www.youtube.com/watch?v=yjNns_R7__Q	Munroe-Meyer Pediatric Feeding Disorders Program Gi and Wyatt
34	https://www.youtube.com/shorts/3wpD0nxsE-E	OPT with Pediatric Dysphagia
35	https://www.youtube.com/shorts/fCaFdBQ4H-0	OPT with Pediatric Dysphagia: The New Breather
36	https://www.youtube.com/watch?v=aveP-nkSfVk	Oral Input | Sensory Processing & Pediatric Occupational Therapy
37	https://www.youtube.com/watch?v=cmAKD1i2S8w	Oral Motor Activities for Feeding Therapy
38	https://www.youtube.com/watch?v=lgmSv--QuiM	OT Rex - Oral Motor and Feeding Development Review
39	https://www.youtube.com/watch?v=JdArBL13h3o	Paediatric Dysphagia - Part 1
40	https://www.youtube.com/watch?v=YZM8pWY_ifc	Paediatric Dysphagia - Part 2
41	https://www.youtube.com/watch?v=gp7muOICILs	Paediatric Dysphagia - Part 3
42	https://www.youtube.com/watch?v=RmRED8dbVyo	Paediatric Dysphagia - Part 4
43	https://www.youtube.com/watch?v=VxcXZiLL_WY	Paeds ENT | Dysphagia in the Paediatric Population | Ms. Rachel Retzler
44	https://www.youtube.com/watch?v=nQ91hfxiZ3Y	Pediatric Center for Airway, Voice and Swallowing Disorders (PCAVS)
45	https://www.youtube.com/watch?v=PTceX2L6A4c	Pediatric Dysphagia
46	https://www.youtube.com/watch?v=uCrfEqpK2uo	Pediatric Dysphagia 620
47	https://www.youtube.com/watch?v=h_jvCmVK5fo	Pediatric Dysphagia Case Study
48	https://www.youtube.com/watch?v=4HHJyRX2_7M	Pediatric Feeding & Swallowing Module IV: Feeding & Swallowing Treatment
49	https://www.youtube.com/watch?v=tpwykR8CLqw	Pediatric feeding and swallowing disorders. By: Prof. Samia Bassiouny
50	https://www.youtube.com/watch?v=NamY7iosuw4	Pediatric Feeding Challenges: It's not just Chewing and Swallowing
51	https://www.youtube.com/watch?v=u5l_HioLg7o	Pediatric feeding disorder identified

52	https://www.youtube.com/watch?v=JVNohD3e2Fo	Pediatric Feeding Disorder Webinar
53	https://www.youtube.com/shorts/9V-qiAspy7U	Perspectives on Pediatric Dysphagia
54	https://www.youtube.com/watch?v=V7ZPrNrA9zU	Surgical Management of Pediatric Dysphagia. Dr. D. Sidell, MD
55	https://www.youtube.com/watch?v=TqH38XT48rY	Therapy Tip of the Week - "First, Then" Feeding Strategy
56	https://www.youtube.com/watch?v=1Gl4I63wq00	Tips for Swallowing Therapy Plateaux | What You Can Do to Improve Your Dysphagia Therapy
57	https://www.youtube.com/watch?v=GpUsVmaRvGw	VFSS Swallowing Study: Videofluoroscopic Swallowing Study
58	https://www.youtube.com/watch?v=8T9J4jvxjZs	What happens in a videofluoroscopy swallow study?
59	https://www.youtube.com/watch?v=X2nGk2DoOt8	What is Feeding Therapy? Sample Session from JCFS' Integrated Pediatric Interventions
60	https://www.youtube.com/watch?v=BRyAZtDCGM0&list=PLksuAcSigteIDJ2qV1m9jcRAUiNRGkTw9&index=4	What the Experts Have to Say - Treatment Approaches
